# Two Targets, One Hit: new Anticancer Therapeutics to Prevent Tumorigenesis Without Cardiotoxicity

**DOI:** 10.3389/fphar.2020.569955

**Published:** 2021-02-10

**Authors:** Zoltán Szabó, Lilla Hornyák, Márton Miskei, Lóránt Székvölgyi

**Affiliations:** ^1^ Department of Emergency Medicine, Faculty of Medicine, University of Debrecen, Debrecen, Hungary; ^2^ MTA-DE Momentum Genome Architecture and Recombination Research Group, Department of Biochemistry and Molecular Biology, Faculty of Medicine, University of Debrecen, Doctoral School of Molecular Cell and Immune Biology, Debrecen, Hungary; ^3^ Faculty of Pharmacy, University of Debrecen, Debrecen, Hungary

**Keywords:** MAPK/ERK pathway, bax, cancer therapy, clinical trial, cardiotoxicity

## Abstract

A serious adverse effect of cancer therapies is cardiovascular toxicity, which significantly limits the widespread use of antineoplastic agents. The promising new field of cardio-oncology offers the identification of potent anti-cancer therapeutics that effectively inhibit cancer cell proliferation without causing cardiotoxicity. Future introduction of recently identified cardio-safe compounds into clinical practice (including ERK dimerization inhibitors or BAX allosteric inhibitors) is expected to help oncologists avoid unwanted cardiological complications associated with therapeutic interventions.

## 1 Introduction

One of the most devastating adverse effects of anticancer treatments is cardiovascular toxicity, which significantly restricts the effective use of conventional and targeted tumor therapeutics (Cameron and Chen, 2018; Herrmann, 2020). Therapy-related cardiovascular diseases involve a wide range of disorders from thromboembolism, stroke, systemic and pulmonary hypertension, myocarditis, cardiac arrhythmias to even sudden cardiac death, which collectively contribute to an increased morbidity and mortality of cancer patients. In addition to cardiovascular damage, a significant fraction of cancer patients suffers from acute or chronic kidney disease, requiring renal replacement therapies (Czifra et al., 2013; Barta et al., 2014), resulting in an increased risk of atrial and ventricular arrhythmias, heart failure and other adverse effects (Szabó et al., 2020). Nevertheless, increased prevalence of cancer among patients with heart failure indicates common molecular triggers and risk factors that contribute to both diseases (de Boer et al., 2019; Meijers and De Boer, 2019). Regardless of hemodynamic impairment, heart failure stimulates tumor development by secreting various circulating factors (Meijers et al., 2018; Richards, 2018), suggesting a causal relationship between heart failure and tumorigenesis. These associations encourage the development of new cardio-safe strategies to increase the effectiveness of cancer therapies (Hetey et al., 2017; Boros-oláh et al., 2019).

**FIGURE 1:**
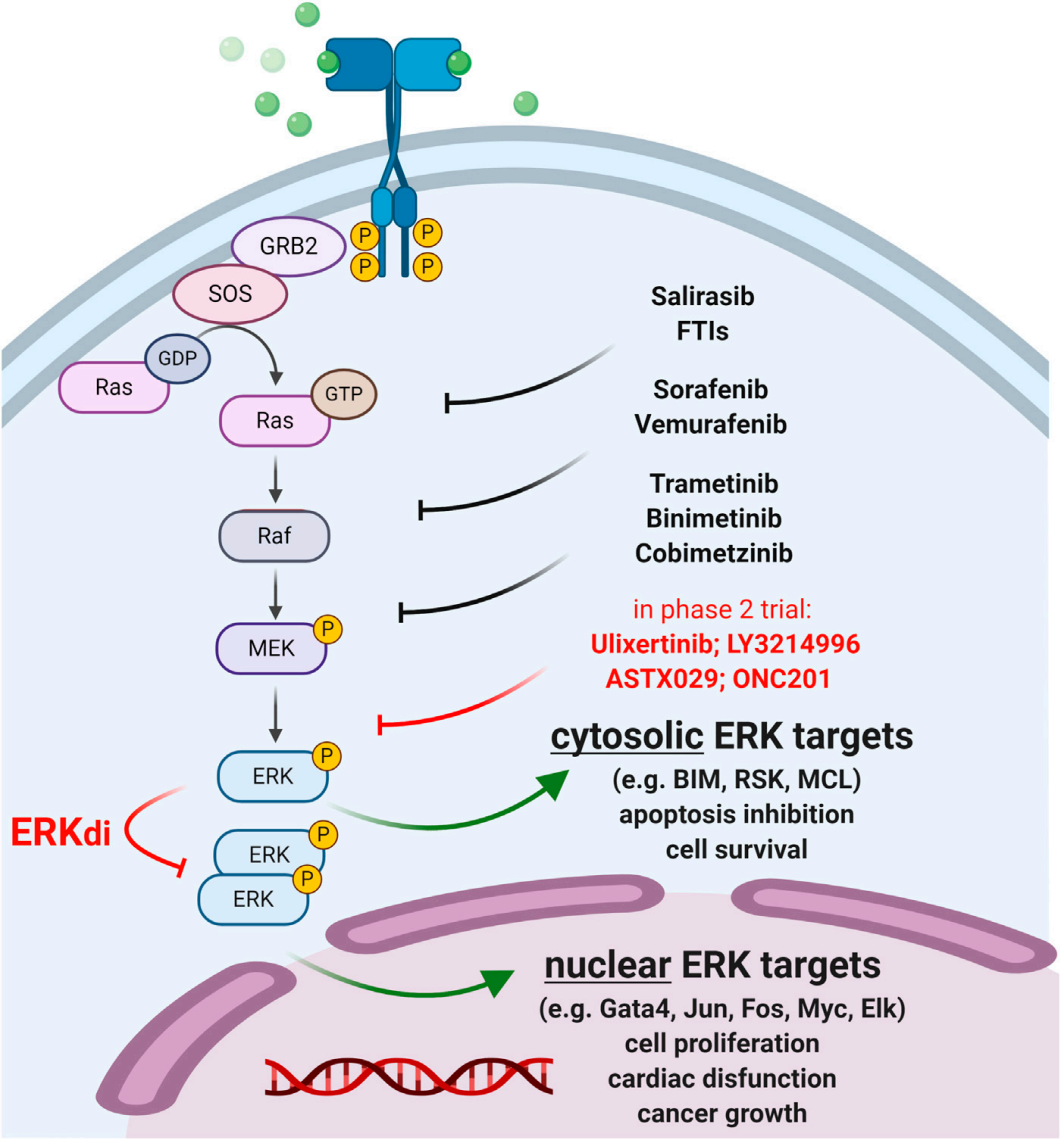
Therapeutic targeting of the MAPK/ERK pathway. Approved anti-cancer compounds targeting RAS/RAF/MEK are indicated in black. ERK inhibitors (ERKi) in phase 2 clinical trials are shown in red. Newly developed ERK dimerization inhibitors (ERKdi) are currently in pre-clinical phases. ERKdi compounds prevent nuclear translocation of ERK, thereby inhibiting the activation of transcription factors that cause tumor cell proliferation and mediate cardiac disfunction in normal cardiac tissue.

The MAPK/ERK pathway is one of the major targets of anti-tumor therapies in several malignancies as gain-of-function mutations of this pathway are collectively associated with more than 90% of cancers (Nissan et al., 2013; Smorodinsky-Atias et al., 2020). The cascade involves the sequential activation of receptor tyrosine kinases (RTK), RAS GTPases, RAF kinases (MAP3K), MEK (MAP2K), which ultimately phosphorylates ERK (MAPK) (Lee et al., 2020). Activated ERK (extracellular signal-regulated kinase) is located at the bottom of the cascade phosphorylating hundreds of cytosolic and nuclear targets (Plotnikov et al., 2015) that mediate opposing signaling pathways leading to cell survival, apoptosis, cancer growth or cardiac disfunction (Figure 1).

Therapeutic targeting of ERK is of key interest since ERK inhibitors (ERKi) have the potential to overcome common drug resistance to “upstream” MEK/RAF/RAS inhibitors (Ryan and Corcoran, 2018; Smorodinsky-Atias et al., 2020). Indeed, ERKi therapies are expected to show greater antitumor efficacy in advanced metastatic and multidrug-resistant tumors with reactivated MAPK signaling, however, their broad clinical use is limited (Lee et al., 2020). This is because ERK is associated with many diseases, such as heart failure, and thus ERKi therapy may cause severe cardiotoxic side effects. Multiple ERK inhibitors are currently in clinical trials for the treatment of RAS/RAF-mutated and BRAFi/MEKi-resistant tumors (Supplementary Table S1); however, new cardio-safe strategies need to be developed to overcome acquired resistance to MAPK inhibitor therapies.

In this respect, recently developed ERK-dimerization inhibitors (ERKdi) show potential to prevent tumorigenesis in preclinical models, without causing severe cardiotoxicity (Herrero et al., 2015; Tomasovic, 2020). The latest compound (Tomasovic, 2020) specifically blocks ERK1/2 dimer formation and consequent Thr188 phosphorylation (that is a prerequisite for ERK nuclear translocation, oncogenic activation and cardiotoxic side effects (Tomasovic, 2020)), preventing heart failure while preserving ERK1/2 catalytic activity and cytosolic survival signaling. This dual consequence of ERKdi treatment is in sharp contrast with the adverse effect of “upstream” MAPK/RAS inhibitors (e.g., MEKi, RAFi), which severely compromise cardiomyocyte mitochondria function and often lead to cardiac injury. The newly developed ERKdi drug may also be relevant in other diseases that rely on intensive ERK signaling, such as secondary cardiac injuries (e.g., due to hypertension, oxidative damage, ischemia), which are expected to extend therapeutic modalities to tumors resistant to upstream MAPK inhibitors.

In addition to MAPK inhibitors, doxorubicin (dox) is one of the most common anthracycline-based chemotherapeutics due to its broad spectrum and high efficacy; however, its applicability is highly limited by its severe cardiotoxic side-effects (Wallace et al., 2020). A recent study identified a new Sirt6–Tip60–Gata4 molecular axis that includes a histone deacetylase (Sirt6), a histone acetylase (Tip60), and a transcription factor (Gata4) that regulates an anti-apoptotic pathway and thus prevents dox-induced cardiotoxicity (Peng et al., 2020). Importantly, Gata4 is activated directly by ERK (via phosphorylation on serine 105 (Liang et al., 2001)) and thus inhibiting the deacetylase activity of Sirt6, which in turn disrupts the equilibrium in local histone acetylation. The resulting open chromatin allows the expression of cardiac-expressed genes involved in cardiac development and hypertrophy, as well as Bcl2 survival factors that suppress apoptosis and dox cardiotoxicity. Therefore, the Sirt6-Tip60-Gata4 axis represents a new therapeutic target to enhance the safety and efficacy of dox chemotherapy.

In another recent attempt, dox-based tumor therapy was made cardio-safe in combination with a specific Bax inhibitor (Amgalan et al., 2020). When dox was administered with a newly developed Bax allosteric inhibitor that modulate Bax function by conformational changes (BAI1 (Garner et al., 2019), Figure 2), cardiomyopathy was completely prevented without compromising the tumor-killing efficacy of dox treatment (Amgalan et al., 2020). The differential effect of Bax inhibition on cardiomyocytes vs. cancerous cells was attributed to the ubiquitously high Bax expression of cancer cells relative to the heart tissue (see e.g., https://www.proteinatlas.org/ENSG00000087088-BAX/pathology; (Thul et al., 2017)). High Bax levels in tumors may help suspend the inhibitory effect of the drug, promoting the apoptosis of cancer cells. These associations designate Bax as a potent tumor-selective drug target to prevent dox-induced cardiotoxicity. While it remains to be tested, the compound may also be used to prevent cardiomyopathies in other diseases in which Bax-mediated apoptotic and necrotic pathways are involved (e.g., infarction, stroke).

**FIGURE 2:**
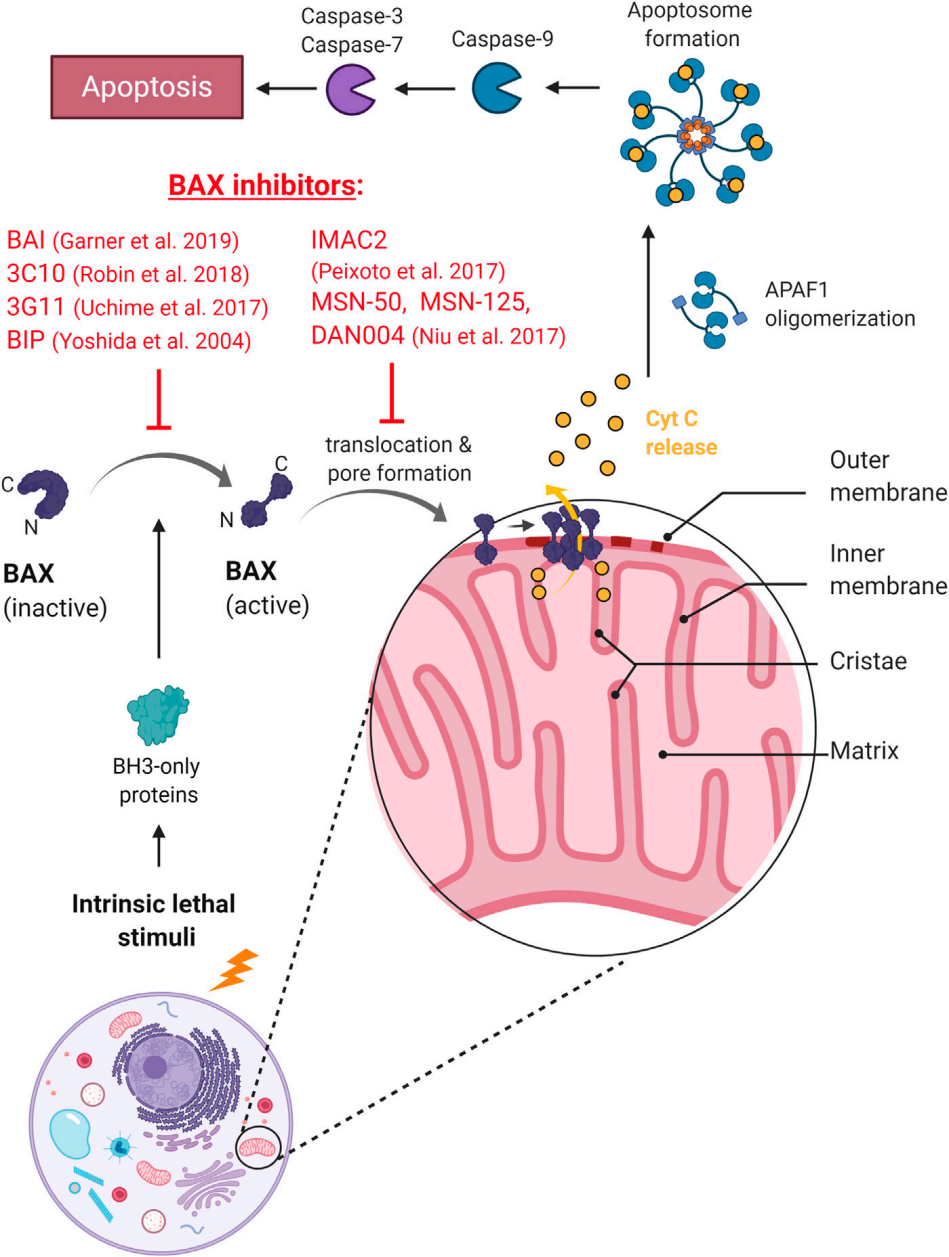
The mechanism of action of BAX inhibitors. BAXi prevents conformational change and activation of BAX, or inhibits mitochondrial translocation and pore formation, preventing the release of cytochrome C and the onset of apoptosis.

## 2 Conclusion

The above cardio-safe drugs (including ERK dimerization inhibitors or BAX allosteric inhibitors) are just the tip of the iceberg: superimposed on them, further research into new molecular pathways and anticancer therapeutics is needed to prevent tumorigenesis without instigating severe cardiotoxicity. BAXi data are still in the preclinical phase and ERKi human clinical trials are still preliminary. Classical ERK inhibitors have some common side effects that can be mild (e.g., diarrhea, nausea, fatigue, and rash) and severe, including neurotoxicity (CC-90003, NCT02313012) and myocardial infarction (Merchant et al., 2014; Sullivan et al., 2018; Chin et al., 2019; Weekes et al., 2020). In addition, tumors treated with ERKi often exhibit resistance to ERK inhibitors, just like upstream Ras/Raf/Mek kinases (Goetz et al., 2014; Jha et al., 2016; Jaiswal et al., 2018), which limits therapeutic efficacy. Whether the new generational ERK dimerization inhibitors (ERKdi) are better tolerable in combination with other cytostatic agents remains to be demonstrated. We also note that the ERK target GATA-4 is regulated by other post-translational modifications in myocardial cells involving e.g. p38 mitogen-activated protein kinase (p38 MAPK), small GTPase RhoA-associated coil-forming kinase (ROCK), glycogen synthase kinase-3beta (GSK-3beta) and cAMP-responsive element-binding protein binding protein (CBP) (Pikkarainen et al., 2004), which greatly increase the complexity of the system in terms of therapeutic options. This is a promising new field of cardio-oncology which is expected to improve the effectiveness of cancer treatments and help physicians avoid unexpected secondary effects related to therapeutic intervention.
