# Supplementary material for: Two Targets, One Hit: new Anticancer Therapeutics to Prevent Tumorigenesis Without Cardiotoxicity
Source: Front Pharmacol. 2021 Feb 10;11:569955. doi: 10.3389/fphar.2020.569955 (PMC7902874; doi:10.3389/fphar.2020.569955)
Supplement: Supplementary file 1 [file table1.docx]

Supplementary Material

# Supplementary Table S1.

# Clinical trials (in phase ≥2) involving ERK inhibitor therapies.

* Note: ONC201 also inhibits Akt.

| **Drug** | **Phase** | **Status** | **Study title** | **URL** |
| --- | --- | --- | --- | --- |
| Ulixertinib (BVD-523) | Phase 2 | Active, not recruiting | A Phase II Study of BVD-523 in Metastatic Uveal Melanoma | <https://ClinicalTrials.gov/show/NCT03417739> |
| Ulixertinib (BVD-523) | Phase 2 | Recruiting | Ulixertinib in Treating Patients With Advanced Solid Tumors, Non-Hodgkin Lymphoma, or Histiocytic Disorders With MAPK Pathway Mutations (A Pediatric MATCH Treatment Trial) | <https://ClinicalTrials.gov/show/NCT03698994> |
| Ulixertinib (BVD-523) | Phase 2 | Recruiting | Targeted Therapy Directed by Genetic Testing in Treating Pediatric Patients With Relapsed or Refractory Advanced Solid Tumors, Non-Hodgkin Lymphomas, or Histiocytic Disorders (The Pediatric MATCH Screening Trial) | <https://ClinicalTrials.gov/show/NCT03155620> |
| Ulixertinib (BVD-523) | Phase 2 | Completed | Phase 1/2 Study of the ERK1/2 Inhibitor BVD-523 in Patients With Acute Myelogenous Leukemia or Myelodysplastic Syndromes | <https://ClinicalTrials.gov/show/NCT02296242> |
| Ulixertinib (BVD-523) | Phase 2 | Completed | Phase I Dose-Escalation, Safety, Pharmacokinetic and Pharmacodynamic Study of BVD-523 in Patients With Advanced Malignancies | <https://ClinicalTrials.gov/show/NCT01781429> |
| ONC201 | Phase 2 | Not yet recruiting | ONC201 and Paclitaxel in Treating Patients With Platinum-Resistant Refractory or Recurrent Epithelial Ovarian, Fallopian Tube, or Primary Peritoneal Cancer | <https://ClinicalTrials.gov/show/NCT04055649> |
| ONC201 | Phase 2 | Recruiting | Phase 2 Study of ONC201 in Neuroendocrine Tumors | <https://ClinicalTrials.gov/show/NCT03034200> |
| ONC201 | Phase 2 | Recruiting | Single Agent ONC201 in Recurrent or Metastatic Endometrial Cancer | <https://ClinicalTrials.gov/show/NCT03099499> |
| ONC201 | Phase 2 | Suspended | ONC201 With and Without Methionine-Restricted Diet in Patients With Metastatic Triple Negative Breast Cancer | <https://ClinicalTrials.gov/show/NCT03733119> |
| ONC201 | Phase 2 | Active, not recruiting | Oral ONC201 in Relapsed/Refractory Multiple Myeloma | <https://ClinicalTrials.gov/show/NCT02863991> |
| ONC201 | Phase 2 | Recruiting | BrUOG 379 Phase Ib/II Trial ONC201 + Nivolumab in MSS mCRC | <https://ClinicalTrials.gov/show/NCT03791398> |
| ONC201 | Phase 2 | Withdrawn | A First-in-man Phase I/II Study of Oral ONC201 in Patients With Advanced Cancer | <https://ClinicalTrials.gov/show/NCT02038699> |
| LY3214996 | Phase 2 | Not yet recruiting | LY3214996 +/- HCQ in Pancreatic Cancer | <https://ClinicalTrials.gov/show/NCT04386057> |
| ASTX029 | Phase 2 | Recruiting | Study of ASTX029 in Subjects With Advanced Solid Tumors | <https://ClinicalTrials.gov/show/NCT03520075> |
